# 

**Published:** 1845-04

**Authors:** 


### CONTENTS

OF THE

### MEDICAL EXAMINER

NEW SERIES—NO. IV.—APRIL, 1845.

---

### ORIGINAL COMMUNICATIONS.

Cancerous communication between the Stomach and Colon. Extract of a letter from Dr. Wm. Waters, of Fredericktown, Maryland. (Communicated by Professor Dunglison,) - - - - - 201

On the Removal of Foreign Bodies from the Meatus Auditorius Externus. By James Bryan, M. D., - - - - - - - 202

### CLINICAL LECTURES AND REPORTS.

PHILADELPHIA HOSPITAL,

*Saturday, January* 4, 1845.

Clinic of Professor Dunglison.

Disorders of the Biliary Secretion, - - - - - - 205

Jaundice, - - - - - - - - - - - 206

Jaundice of the Infant, - - - - - - - 211

### BIBLIOGRAPHICAL NOTICES.

Elementary Treatise on Internal Pathology. By MM. A. Hardy and J. Behier, - - - - - - - - - - - 212

Elementary and Practical Treatise on Internal Pathology. By A. Grisolle, &c. &c., - - - - - - - - - - 212

Dictionary of Medicine, or General Repertory of the Medical Sciences, considered theoretically and practically. By MM. Adelon, Béclard, &c., - - - - - - - - - - 212

Sixth Annual Report of the Directors and Superintendent of the Ohio Lunatic Asylum to the Forty-third General Assembly, December 9, 1844, - - - - - - - - - - - 220

Reports of the Trustees, Steward, Treasurer and Superintendant of the Insane Hospital, 1844. Augusta, (Me.,) 1845, - - - 220

Report of the Pennsylvania Hospital for the Insane for the year 1844. By Thomas S. Kirkbride, M. D., Physician to the Institution, - 220

A Practical Treatise on the Diseases peculiar to Women, illustrated with Cases, derived from Hospital and Private Practice. By Samuel Ashwell, M. D. Member of the Royal College of Physicians, London, and Obstetric Physician and Lecturer to Guy's Hospital. First American from the last London edition. With notes, by Paul B. Goddard, M. D., M. A. P. S., M. A. N. S., Lecturer on Anatomy, &c., - - - - - - - - - - 225

A Treatise on the Diseases and Special Hygiéne of Females. By Colombat De L'Isére. Translated from the French, with Additions. By Charles D. Meigs, M. D., Professor of Midwifery and the Diseases of Women and Children in Jefferson Medical College, Philadelphia, &c. &c., - - - - - - - - - 225

The Principles of Surgery. By James Miller, F. R. S. E., F. R. C. S. E., Professor of Surgery in the University of Edinburgh, Surgeon to the Royal Infirmary, &c. &c., - - - - - - - 228

Remarks on the influence of Mental Cultivation and Mental Excitement upon Health. By Amariah Brigham, M. D., Superintendant and Physician to the State Lunatic Asylum, Utica, New York, - 230

The Sanatory condition of the Laboring population of New York, with suggestions for its improvement; a Discourse (with additions) delivered on the 30th of December, 1844, at the Repository of the American Institute. By John H. Griscom, M. D., Fellow of the College of Physicians and Surgeons; Physician of the New York Hospital; late Physician of the City and Eastern Dispensaries, - 230

A Dictionary of Practical Medicine, &c. By James Copland, M. D., F. R. S. Edited by Charles A. Lee, M. D., - - - - 233

Twenty-fourth Annual Report of the Bloomingdale Asylum for the Insane. By Pliny Earl, M. D., Physician to the Institution, - 233

### EDITORIAL.

To Correspondents and Publishers, - - - - - - - 233

Mesmerism. - - - - . - - - - - - 233

Medical and Chirurgical Society of London, - - - - - 235

### RECORD OF MEDICAL SCIENCE.

Medical Society of London, Jan. 13, 1845.

Hooping-Cough, its Treatment and Effects, - - - - 235

Belladonna in Hooping-Cough, - - - - - - - 235

Blisters for Children, - - - - - - - 237

Syrian Medical Association, Hospital at Damascus, - - - - 237

Rupture of the Uterus and Abdominal Parieties. Cæsarean Operation performed with Success, both for the Mother and Infant. Thirteen months afterwards, Rupture of the Uterus and Abdominal Parietes, from a second Pregnancy; Birth of the Infant by this spontaneous Opening, and perfect Recovery of the Mother. By Dr. Prael, Director of the Obstetrical Institution of the city of Hildesheim. - - - - - - - - - - 238

Case of Colloid Tumor in the Cavity of the Cranium, Presented to the Medical Department of the National Institute at its Meeting on the first of July, 1844. By Thomas Miller, M.D., - - 242

On the formation of the Buffy Coat of the Blood. By George Gulliver, F. R.S., Surgeon in the Royal Regiment of Horse Guards, - 246
Observations on the Relaxed Rectum. By H. Hunt, M. D., . - 247
Researches on the Ovum and Corpus Luteum of Man and the lower Animals. By M. Deschamps, - - - - - - - - - 250
Effects of extirpation of the Spleen and Thyroid Gland. By M. Bardeleben, - - - - - - - - - - - - - 250
Prognosis of Chancre with reference to the probabilities of Secondary Symptoms. By M. Ricord, of Paris, - - - - - - 251
Copaiba more active when combined with Purgatives. By M. Jacquetant, - - - - - - - - - - - - - - - 251
Extirpation of the Lachrymal Gland for the cure of Fistula Lachrymalis. By M. Paul Bernard, - - - - - - - - - - 252
Cases of Premature Delivery, - - - - - - - - - - 252
Auto-Plastic Operation for the Cure of Ectropium, - - - - 252
A Fish-hook removed from the Œsophagus without an operation. Reported by Andrew R. Kilpatrick, M. D., Woodville, Miss., - 254
Case of Occlusion of the Vagina in a pregnant woman. By A. Davizac, M. D., of New Orleans, - - - - - - - - - - 255
Ligature of a Polypus of the Bladder, - - - - - - - 257
Sheffield Medical Society, December 12, 1844.
Enlargement of the Heart; Diseased Valves; Hemiplegia. - - 257
Enlargement of the Breasts in a Male; atrophy of the Genital Organs, - - - - - - - - - - - - - 258
Death of a Surgeon from Prussic Acid, - - - - - - - 258
Westminster Medical Society, Feb. 1, 1845.
Foreign Bodies in the System, - - - - - - - - 259

